# [(1,2,5,6-η)-Cyclo­octa-1,5-diene](1-methyl-3-propylimidazol-2-yl­idene-κ*C*)(tri­phenyl­phosphane-κ*P*)iridium(I) tetra­fluorido­borate

**DOI:** 10.1107/S2414314626008527

**Published:** 2026-08-25

**Authors:** Benedikt Kienle, Michael Gau, Daniel R. Albert, Edward Rajaseelan

**Affiliations:** a Lancaster Country Day School, 725 Hamilton Road, Lancaster, PA 17603, USA; b Department of Chemistry, University of Pennsylvania, Philadelphia, PA 19104, USA; c Department of Chemistry Millersville University,Millersville PA 17551 USA; Vienna University of Technology, Austria

**Keywords:** crystal structure, N-heterocyclic carbene, COD ligand, iridium complex, disorder

## Abstract

A new imidazole-based N-heterocyclic carbene iridium(I) cationic complex with a tetra­fluorido­borate counter-anion, [Ir(C_8_H_12_)(C_7_H_12_N_2_)(C_18_H_15_P)]BF_4_, has been synthesized and structurally characterized. The complex cation has a distorted square-planar conformation around the Ir^I^ atom, formed by a bidentate cyclo­octa-1,5-diene (COD) ligand, a phosphane ligand, and an N-heterocyclic carbene (NHC) ligand. The complex crystallizes in the triclinic space group *P*1 with one cationic Ir-NHC complex and one disordered [BF_4_]^−^ anion in the asymmetric unit. Bond lengths and bond angles are as expected for an Ir-NHC complex. Several non-classical C—H⋯F hydrogen-bonding inter­actions between the various ligands (tri­phenyl­phosphane, NHC, and COD) and the tetra­fluorido­borate anion orient the metal cationic complex and the counter-anion in the crystal structure.

## Abstract 2

The complex cation in the title compound has a distorted square-planar conformation around the Ir^I^ atom, formed by a bidentate cyclo­octa-1,5-diene (COD) ligand, a phosphane ligand, and an N-heterocyclic carbene (NHC) ligand.

## Graphical abstract

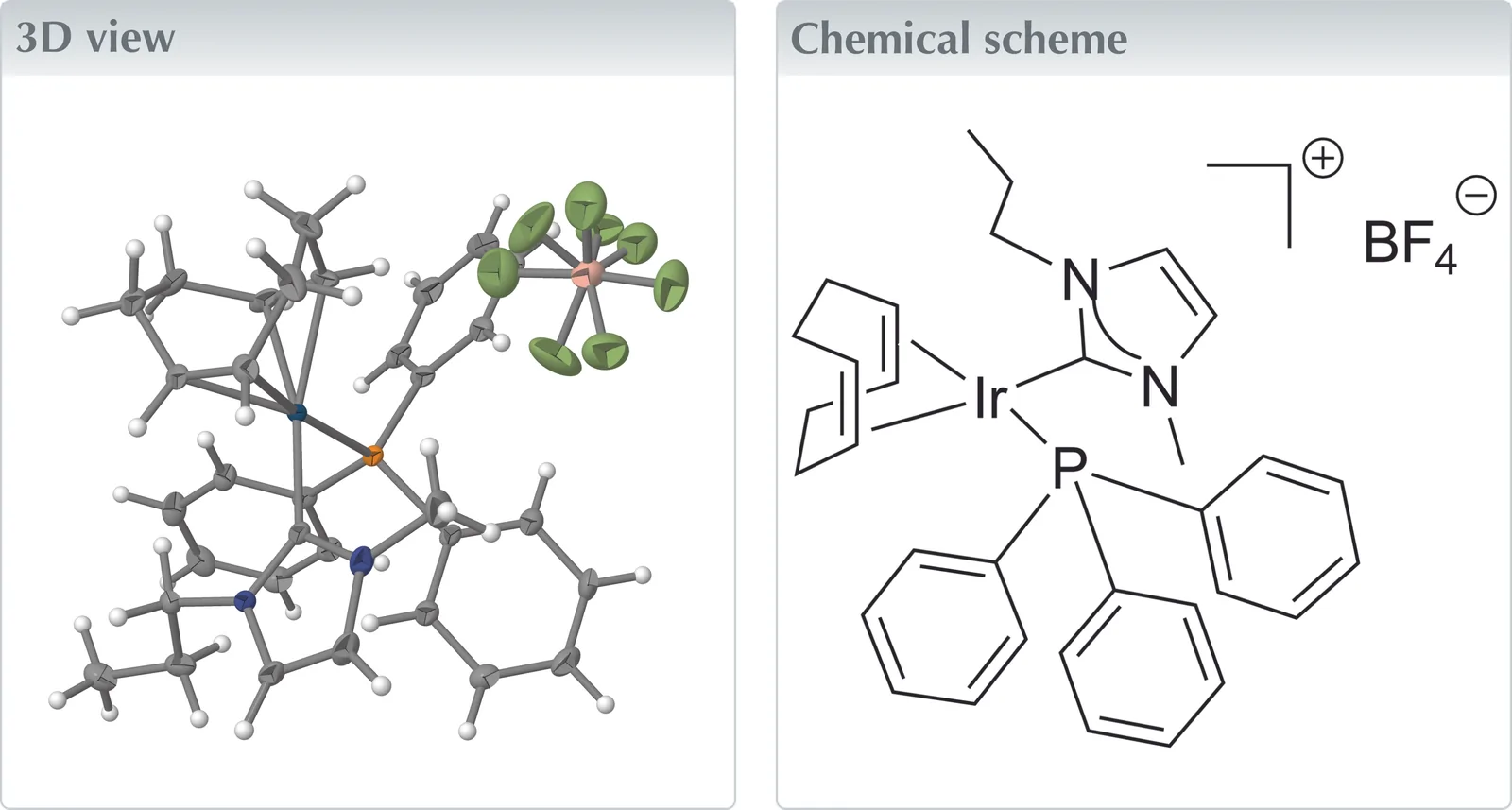

## Structure description

N-heterocyclic carbenes (NHCs) have emerged as excellent alternative ligands for phosphines in transition metal complexes in the study of homogeneous catalysis (Cazin, 2013 ▸; de Frémont *et al.*, 2009 ▸; Díez-González *et al.*, 2009 ▸; Rovis & Nolan, 2013 ▸; Ruff *et al.*, 2016 ▸; Zuo *et al.*, 2014 ▸). Their catalytic activity in the transfer hydrogenation of unsaturated double bonds, especially in ketones and imines, has also been studied and reported (Albrecht *et al.*, 2002 ▸; Gnanamgari *et al.*, 2007 ▸). The NHC ligands can be tuned sterically and electronically by having different substituents (wing tips) on the nitro­gen atoms (Gusev, 2009 ▸). We continue to synthesize new imidazole- and triazole-based NHC complexes of rhodium and iridium, to study the catalytic effect of different substituents on the NHCs and have reported the syntheses, structures, and intra- and inter-mol­ecular hydrogen-bonding inter­actions in several complexes with tri­phenyl­phosphane as an ancillary ligand (Nichol *et al.*, 2009 ▸, 2011 ▸, 2012 ▸; Idrees *et al.*, 2017 ▸; Rood *et al.*, 2021 ▸; Newman *et al.*, 2021 ▸; Castaldi *et al.*, 2021 ▸; Maynard *et al.*, 2023 ▸; Lerch *et al.*, 2024*a* ▸,*b* ▸, 2025 ▸). Here we report the crystal structure of a new Ir^I^ NHC complex with a [BF_4_]^−^ counter-anion, [Ir(C_8_H_12_)(C_7_H_12_N_2_)(C_18_H_15_P)][BF_4_] (**2**).

**Figure 1:**
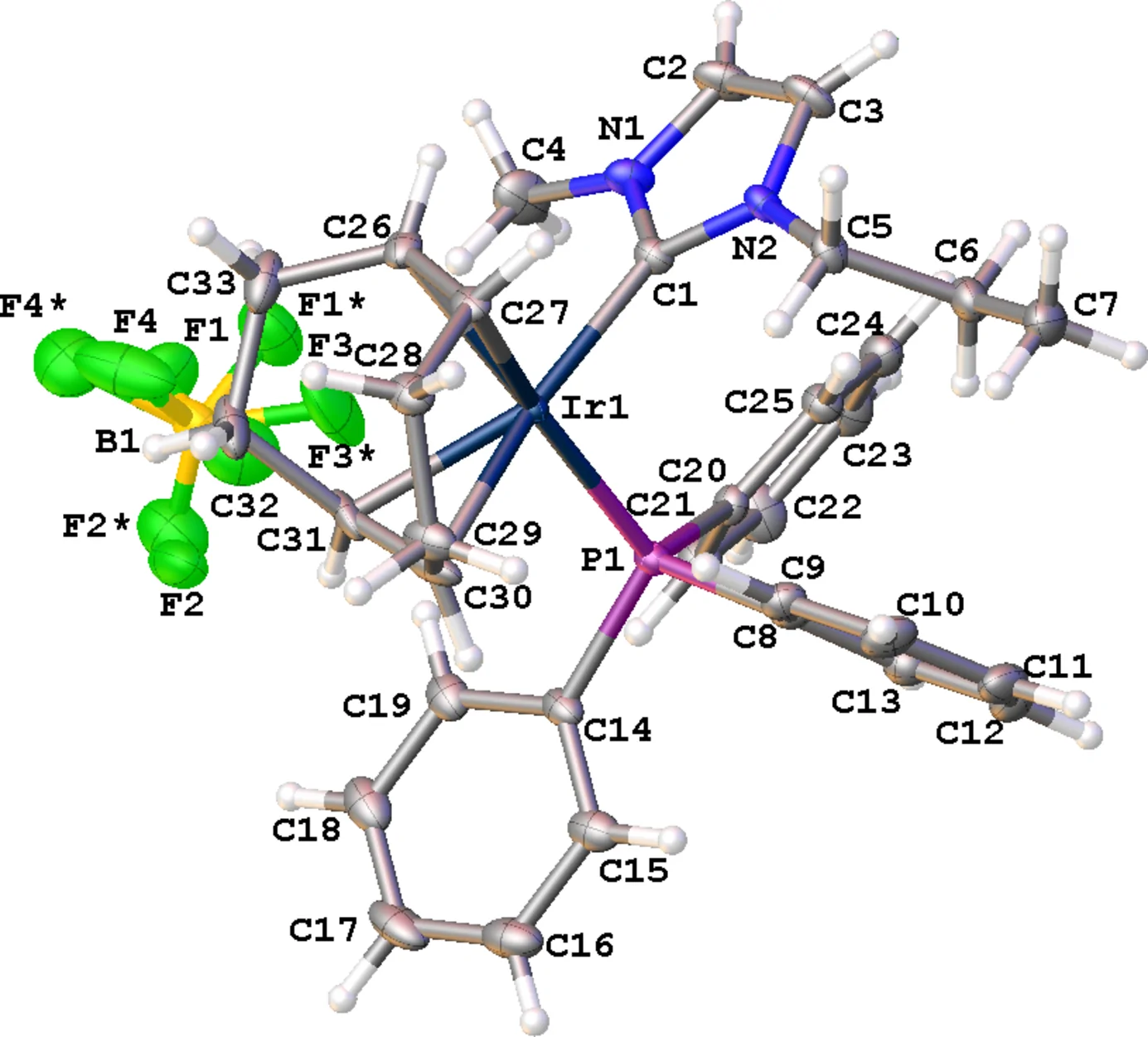
Mol­ecular entities in the crystal structure of the title compound (**2**). Displacement ellipsoids are drawn at the 50% probability level.

**Figure 2:**
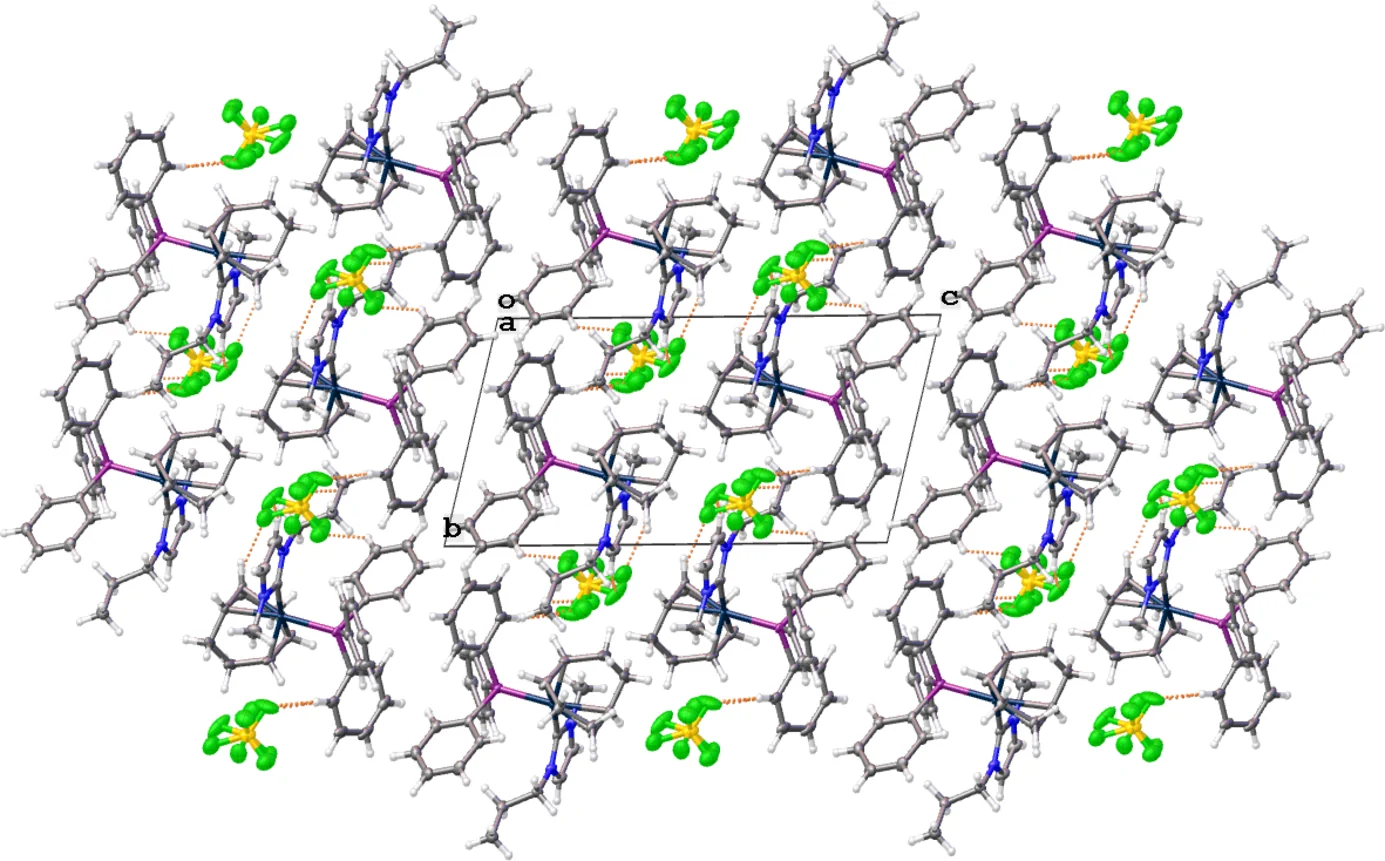
Packing diagram of the title compound (**2**) viewed along the *a* axis. Non-classical hydrogen-bonding inter­actions between the the cationic metal complex ligands and the tetra­fluorido­borate anions are shown as dashed orange lines.

The mol­ecular entities of (**2**) are illustrated in Fig. 1 ▸. No solvent mol­ecules were found in the structure. The coordination sphere around the Ir^I^ cation, formed by the bidentate cyclo­octa-1,5-diene (COD), the NHC, and the tri­phenyl­phosphane ligands, results in a distorted square-planar conformation, characterized by a C1_NHC_—Ir1—P1 bond angle of 91.96 (6)°. The C1 atom of the NHC ligand deviates from the expected *sp*^2^ hybridization in that the N1—C1—N2 bond angle in the imidazole-based carbene is 104.6 (2)°. Other selected bond lengths in the structure are: Ir1— C1_(NHC)_ = 2.045 (2) Å and Ir1— P1 = 2.3190 (6) Å. Fig. 2 ▸ shows the packing diagram of the crystal structure with non-classical C—H⋯F inter­actions displayed as dashed orange lines. The close F⋯H contacts and other numerical data of these inter­actions are summarized in Table 1 ▸. The short anomalous C—H⋯F inter­actions are likely artefacts of the positional disorder associated with the [BF_4_]^−^ anion. The cationic units pack with triphenyl phosphane ligands oriented towards each other on adjacent metal complexes. However, no substantial C—H⋯π inter­actions were found between the phenyl moieties.

## Synthesis and crystallization

**Figure 3:**
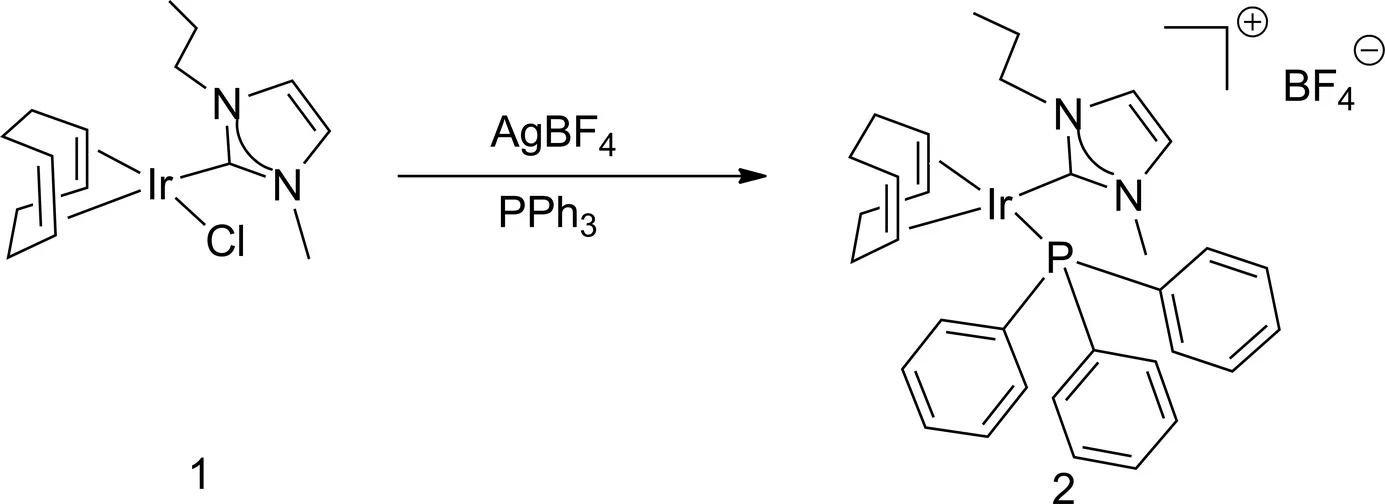
Reaction scheme for the synthesis of the title compound (**2**)

The synthesis of **[(1,2,5,6-η)-cyclo­octa-1,5-diene] (1-methyl-3-propylimidazol-2-yl­idene)chlorido­iridium (1)** has been published previously (Kienle *et al.*, 2026 ▸). All other compounds used in the syntheses, summarized in Fig. 3 ▸, were obtained from Sigma-Aldrich and used as received. NMR spectra were recorded at room temperature in CDCl_3_ on a 400 MHz Varian spectrometer (operating at 100 MHz for ^13^C and 162 MHz for ^31^P) and referenced to the residual solvent peak (δ in p.p.m.).

**[(1,2,5,6-η)-Cyclo­octa-1,5-diene] (1-methyl-3-propylimid­azol-2-yl­idene)(tri­phenyl­phosphine)iridium(I) tetra­fluorido­borate (2):** Tri­phenyl­phosphane (0.075 g, 0.287 mmol) and AgBF_4_ (0.056 g, 0.287 mmol) were added to (**1**) (0.132 g, 0.287 mmol) in CH_2_Cl_2_ (15 ml). The solution was stirred in the dark for 1.5 h under a nitro­gen atmosphere. The resulting mixture was filtered through Celite, and the solvent was removed under reduced pressure. The bright orange–red solid product (**2**) was dried under vacuum. Yield: 0.219 g (99.5%). Orange–red crystals suitable for data collection were obtained by slow diffusion of pentane into a CH_2_Cl_2_ solution. ^1^H NMR: δ 7.49–7.21 (*m*, 15H, H_phen­yl_), 7.04 (*d*, 1H, N—C4H), 6.94 (*d*, 1H, N—C5H), 5.29 (*s*, 3H, N—CH_3_), 4.45–4.29 (*m*, 4 H, CH of COD), 3.49 (*t*, 2H, N–CH_2_ of prop­yl), 2.37 (*m*, 2 H, CH_2_ of prop­yl), 2.28 (*m*, 2H, CH_2_ of COD), 2.03–1.94 (*m*, 2H, CH_2_ of COD), 1.64–1.58 (*m*, 2H, CH_2_ of COD), 1.37–1.33 (*m*, 2H, CH_2_ of COD), 0.88 (*t*, 3 H, CH_3_ of prop­yl). ^13^C NMR: δ 173.06 (Ir—C), 133.62 (N—C4H), 133.52 (N—C5H), 131.28–128.92 (C_phen­yl_), 86.69, 86.58, 85.49, 85.37 (CH of COD), 51.91 (N—CH_3_), 37.23 (N—CH_2_ of prop­yl), 31.54, 31.04, 31.01, 30.85 (CH_2_ of COD), 23.00 (CH_2_ of prop­yl), 11.36 (CH_3_ of prop­yl). ^31^P NMR: δ 18.38.

## Refinement

Crystal data, data collection, and structure refinement details are summarized in Table 2 ▸. The F atoms of the [BF_4_]^−^ anion are disordered over two sets of sites in a refined 0.893 (3):0.107 (3) ratio (minor component labelled with asterisks). For modelling the disorder, B—F bond lengths and F—B—F angles were restrained to reasonable values, and the anisotropic displacement parameters of the B and F atoms to be similar.

## Supplementary Material

Crystal structure: contains datablock(s) I. DOI: 10.1107/S2414314626008527/wm4257sup1.cif

Structure factors: contains datablock(s) I. DOI: 10.1107/S2414314626008527/wm4257Isup2.hkl

CCDC reference: 2581838

Additional supporting information:  crystallographic information; 3D view; checkCIF report

## Figures and Tables

**Table 1:** Hydrogen-bond geometry (Å, °)

*D*—H⋯*A*	*D*—H	H⋯*A*	*D*⋯*A*	*D*—H⋯*A*
C3—H3⋯F4^i^	0.95	2.38	3.100 (4)	133
C5—H5*A*⋯F1^ii^	0.99	2.46	3.265 (3)	138
C7—H7*B*⋯F1*^ii^	0.98	2.53	3.263 (13)	132
C10—H10⋯F2*^ii^	0.95	2.31	3.073 (16)	136
C19—H19⋯F3	0.95	2.47	3.413 (4)	171
C19—H19⋯F3*	0.95	2.53	3.272 (16)	135
C28—H28*B*⋯F4*^ii^	0.99	2.37	3.292 (18)	154

Symmetry codes: (i) 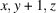; (ii) 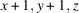.

**Table 2:** Experimental details

Crystal data	
Chemical formula	[Ir(C_8_H_12_)(C_7_H_12_N_2_)(C_18_H_15_P)]BF_4_
*M*_r_	773.64
Crystal system, space group	Triclinic, *P* 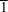
Temperature (K)	100
*a*, *b*, *c* (Å)	9.9795 (2), 10.2191 (2), 17.4844 (4)
α, β, γ (°)	98.173 (2), 98.654 (2), 116.906 (2)
*V* (Å^3^)	1526.60 (6)
*Z*	2
Radiation type	Mo *K*α
μ (mm^−1^)	4.48
Crystal size (mm)	0.13 × 0.1 × 0.08

Data collection	
Diffractometer	Rigaku XtaLAB Synergy-S
Absorption correction	Multi-scan (*CrysAlis PRO*; Rigaku OD, 2025 ▸)
*T*_min_, *T*_max_	0.719, 1.000
No. of measured, independent and observed [*I* > 2σ(*I*)] reflections	38499, 7566, 6931
*R*_int_	0.047
(sin θ/λ)_max_ (Å^−1^)	0.667

Refinement	
*R*[*F*^2^ > 2σ(*F*^2^)], *wR*(*F*^2^), *S*	0.023, 0.048, 1.07
No. of reflections	7566
No. of parameters	418
No. of restraints	116
H-atom treatment	H-atom parameters constrained
Δρ_max_, Δρ_min_ (e Å^−3^)	0.89, −0.85

Computer programs: *CrysAlis PRO* (Rigaku OD, 2025 ▸), *SHELXT* (Sheldrick, 2015*a* ▸), *SHELXL* (Sheldrick, 2015*b* ▸), *OLEX2* (Dolomanov *et al.*, 2009 ▸) and *publCIF* (Westrip, 2010 ▸).

## full crystallographic data

### [(1,2,5,6-η)-Cycloocta-1,5-diene](1-methyl-3-propylimidazol-2-ylidene-κC)(triphenylphosphane-κP)iridium(I) tetrafluoridoborate Crystal data

**Table:**

[Ir(C_8_H_12_)(C_7_H_12_N_2_)(C_18_H_15_P)]BF_4_	*Z* = 2
*M**_r_* = 773.64	*F*(000) = 768
Triclinic, *P*1	*D*_x_ = 1.683 Mg m^−^^3^
*a* = 9.9795 (2) Å	Mo *K*α radiation, λ = 0.71073 Å
*b* = 10.2191 (2) Å	Cell parameters from 27360 reflections
*c* = 17.4844 (4) Å	θ = 3.7–28.3°
α = 98.173 (2)°	µ = 4.48 mm^−^^1^
β = 98.654 (2)°	*T* = 100 K
γ = 116.906 (2)°	Block, red
*V* = 1526.60 (6) Å^3^	0.13 × 0.1 × 0.08 mm

### [(1,2,5,6-η)-Cycloocta-1,5-diene](1-methyl-3-propylimidazol-2-ylidene-κC)(triphenylphosphane-κP)iridium(I) tetrafluoridoborate Data collection

**Table:**

Rigaku XtaLAB Synergy-S diffractometer	7566 independent reflections
Radiation source: micro-focus sealed X-ray tube, PhotonJet (Mo) X-ray Source	6931 reflections with *I* > 2σ(*I*)
Mirror monochromator	*R*_int_ = 0.047
Detector resolution: 10.0 pixels mm^-1^	θ_max_ = 28.3°, θ_min_ = 3.4°
ω scans	*h* = −13→13
Absorption correction: multi-scan (CrysAlisPro; Rigaku OD, 2025)	*k* = −13→13
*T*_min_ = 0.719, *T*_max_ = 1.000	*l* = −23→20
38499 measured reflections	

### [(1,2,5,6-η)-Cycloocta-1,5-diene](1-methyl-3-propylimidazol-2-ylidene-κC)(triphenylphosphane-κP)iridium(I) tetrafluoridoborate Refinement

**Table:**

Refinement on *F*^2^	116 restraints
Least-squares matrix: full	Hydrogen site location: inferred from neighbouring sites
*R*[*F*^2^ > 2σ(*F*^2^)] = 0.023	H-atom parameters constrained
*wR*(*F*^2^) = 0.048	*w* = 1/[σ^2^(*F*_o_^2^) + (0.0216*P*)^2^] where *P* = (*F*_o_^2^ + 2*F*_c_^2^)/3
*S* = 1.07	(Δ/σ)_max_ = 0.006
7566 reflections	Δρ_max_ = 0.89 e Å^−^^3^
418 parameters	Δρ_min_ = −0.85 e Å^−^^3^

### [(1,2,5,6-η)-Cycloocta-1,5-diene](1-methyl-3-propylimidazol-2-ylidene-κC)(triphenylphosphane-κP)iridium(I) tetrafluoridoborate Special details

**Table:**

Geometry. All esds (except the esd in the dihedral angle between two l.s. planes) are estimated using the full covariance matrix. The cell esds are taken into account individually in the estimation of esds in distances, angles and torsion angles; correlations between esds in cell parameters are only used when they are defined by crystal symmetry. An approximate (isotropic) treatment of cell esds is used for estimating esds involving l.s. planes.

### [(1,2,5,6-η)-Cycloocta-1,5-diene](1-methyl-3-propylimidazol-2-ylidene-κC)(triphenylphosphane-κP)iridium(I) tetrafluoridoborate Fractional atomic coordinates and isotropic or equivalent isotropic displacement parameters (Å^2^)

**Table:**

	*x*	*y*	*z*	*U*_iso_*/*U*_eq_	Occ. (<1)
Ir1	0.76080 (2)	0.69914 (2)	0.33134 (2)	0.01191 (4)	
P1	0.63652 (7)	0.62806 (7)	0.19739 (3)	0.01392 (13)	
N1	0.5302 (2)	0.7864 (3)	0.38118 (12)	0.0194 (5)	
N2	0.7153 (2)	0.9834 (2)	0.36070 (12)	0.0156 (4)	
C1	0.6652 (3)	0.8347 (3)	0.35723 (14)	0.0150 (5)	
C2	0.4973 (3)	0.9029 (3)	0.39781 (16)	0.0256 (6)	
H2	0.409527	0.896922	0.415274	0.031*	
C3	0.6117 (3)	1.0266 (3)	0.38492 (16)	0.0227 (6)	
H3	0.620197	1.124136	0.391127	0.027*	
C4	0.4317 (3)	0.6318 (3)	0.38522 (18)	0.0303 (7)	
H4A	0.337925	0.584613	0.341694	0.045*	
H4B	0.402910	0.631872	0.436387	0.045*	
H4C	0.487942	0.574619	0.380290	0.045*	
C5	0.8579 (3)	1.0886 (3)	0.34183 (15)	0.0179 (5)	
H5A	0.917385	1.035979	0.329750	0.021*	
H5B	0.922411	1.173715	0.388969	0.021*	
C6	0.8255 (3)	1.1505 (3)	0.27110 (16)	0.0235 (6)	
H6A	0.748759	1.184585	0.278083	0.028*	
H6B	0.780647	1.069267	0.221674	0.028*	
C7	0.9734 (3)	1.2826 (3)	0.26315 (17)	0.0284 (6)	
H7A	0.949809	1.318063	0.216307	0.043*	
H7B	1.050050	1.249495	0.257052	0.043*	
H7C	1.015166	1.365146	0.310971	0.043*	
C8	0.7542 (3)	0.7710 (3)	0.14884 (14)	0.0165 (5)	
C9	0.9122 (3)	0.8643 (3)	0.18480 (14)	0.0191 (5)	
H9	0.956694	0.848910	0.232347	0.023*	
C10	1.0048 (3)	0.9790 (3)	0.15201 (15)	0.0257 (6)	
H10	1.112216	1.040438	0.176447	0.031*	
C11	0.9399 (3)	1.0033 (3)	0.08368 (16)	0.0268 (6)	
H11	1.001896	1.084313	0.062264	0.032*	
C12	0.7843 (3)	0.9098 (3)	0.04617 (16)	0.0255 (6)	
H12	0.741305	0.925034	−0.001868	0.031*	
C13	0.6907 (3)	0.7940 (3)	0.07837 (15)	0.0214 (6)	
H13	0.584177	0.730871	0.052591	0.026*	
C14	0.6156 (3)	0.4511 (3)	0.14282 (14)	0.0186 (5)	
C15	0.6963 (3)	0.4461 (3)	0.08455 (15)	0.0237 (6)	
H15	0.752636	0.534566	0.066772	0.028*	
C16	0.6945 (3)	0.3125 (4)	0.05244 (16)	0.0302 (7)	
H16	0.749645	0.310053	0.012939	0.036*	
C17	0.6124 (3)	0.1833 (4)	0.07803 (17)	0.0346 (7)	
H17	0.613074	0.092710	0.056839	0.042*	
C18	0.5291 (3)	0.1856 (3)	0.13458 (16)	0.0288 (7)	
H18	0.471009	0.095995	0.151082	0.035*	
C19	0.5306 (3)	0.3180 (3)	0.16694 (15)	0.0224 (6)	
H19	0.473747	0.318899	0.205783	0.027*	
C20	0.4438 (3)	0.6129 (3)	0.16866 (14)	0.0159 (5)	
C21	0.3129 (3)	0.4778 (3)	0.12636 (15)	0.0221 (6)	
H21	0.321557	0.390056	0.109329	0.026*	
C22	0.1686 (3)	0.4725 (3)	0.10920 (17)	0.0290 (6)	
H22	0.079177	0.380791	0.080392	0.035*	
C23	0.1554 (3)	0.6005 (4)	0.13401 (16)	0.0270 (6)	
H23	0.056806	0.595390	0.122892	0.032*	
C24	0.2851 (3)	0.7352 (3)	0.17481 (15)	0.0221 (6)	
H24	0.276205	0.823025	0.191176	0.027*	
C25	0.4292 (3)	0.7410 (3)	0.19177 (14)	0.0190 (5)	
H25	0.518610	0.833737	0.219469	0.023*	
C26	0.8276 (3)	0.7324 (3)	0.46130 (14)	0.0187 (5)	
H26	0.783435	0.786646	0.492136	0.022*	
C27	0.9567 (3)	0.8266 (3)	0.43557 (13)	0.0159 (5)	
H27	0.985996	0.935821	0.451127	0.019*	
C28	1.0940 (3)	0.8015 (3)	0.43040 (15)	0.0221 (6)	
H28A	1.104109	0.742895	0.469289	0.026*	
H28B	1.189739	0.900528	0.445105	0.026*	
C29	1.0785 (3)	0.7170 (3)	0.34699 (15)	0.0246 (6)	
H29A	1.125587	0.791568	0.315423	0.029*	
H29B	1.137213	0.660995	0.351788	0.029*	
C30	0.9123 (3)	0.6070 (3)	0.30276 (16)	0.0244 (6)	
H30	0.897675	0.569995	0.244435	0.029*	
C31	0.8005 (3)	0.5068 (3)	0.33581 (16)	0.0241 (6)	
H31	0.720534	0.411240	0.296654	0.029*	
C32	0.8340 (4)	0.4887 (3)	0.42047 (16)	0.0321 (7)	
H32A	0.773136	0.380822	0.420775	0.039*	
H32B	0.945144	0.518879	0.437934	0.039*	
C33	0.7949 (3)	0.5822 (3)	0.47905 (16)	0.0302 (7)	
H33A	0.683774	0.524259	0.478301	0.036*	
H33B	0.855650	0.599996	0.533355	0.036*	
F1	0.1906 (2)	0.0948 (3)	0.35489 (13)	0.0458 (6)	0.893 (3)
F1*	0.2577 (17)	0.2470 (18)	0.3585 (10)	0.049 (3)	0.107 (3)
F2	0.3576 (3)	0.0864 (3)	0.28345 (12)	0.0409 (6)	0.893 (3)
F2*	0.283 (2)	0.0588 (16)	0.2883 (9)	0.050 (4)	0.107 (3)
F3	0.3455 (3)	0.3005 (3)	0.31721 (18)	0.0642 (8)	0.893 (3)
F3*	0.4904 (13)	0.2830 (17)	0.3464 (10)	0.056 (3)	0.107 (3)
F4	0.4457 (3)	0.2159 (4)	0.41373 (15)	0.0630 (9)	0.893 (3)
F4*	0.368 (2)	0.131 (2)	0.4202 (8)	0.050 (4)	0.107 (3)
B1	0.3370 (4)	0.1759 (4)	0.3448 (2)	0.0318 (7)	

### [(1,2,5,6-η)-Cycloocta-1,5-diene](1-methyl-3-propylimidazol-2-ylidene-κC)(triphenylphosphane-κP)iridium(I) tetrafluoridoborate Atomic displacement parameters (Å^2^)

**Table:**

	*U*^11^	*U*^22^	*U*^33^	*U*^12^	*U*^13^	*U*^23^
Ir1	0.01190 (5)	0.01026 (5)	0.01223 (5)	0.00581 (4)	−0.00028 (3)	0.00064 (3)
P1	0.0127 (3)	0.0138 (3)	0.0133 (3)	0.0069 (3)	−0.0004 (2)	0.0000 (2)
N1	0.0132 (10)	0.0203 (12)	0.0219 (11)	0.0069 (9)	0.0051 (8)	0.0001 (9)
N2	0.0130 (9)	0.0122 (11)	0.0197 (10)	0.0068 (9)	0.0006 (8)	0.0000 (8)
C1	0.0118 (11)	0.0153 (13)	0.0157 (11)	0.0067 (10)	−0.0004 (9)	0.0001 (10)
C2	0.0143 (12)	0.0282 (16)	0.0314 (15)	0.0116 (12)	0.0044 (10)	−0.0046 (12)
C3	0.0157 (12)	0.0207 (15)	0.0309 (14)	0.0124 (11)	0.0006 (10)	−0.0035 (11)
C4	0.0181 (13)	0.0267 (17)	0.0409 (17)	0.0051 (12)	0.0122 (12)	0.0081 (13)
C5	0.0145 (11)	0.0130 (13)	0.0230 (13)	0.0051 (10)	0.0017 (9)	0.0036 (10)
C6	0.0288 (14)	0.0218 (15)	0.0229 (13)	0.0158 (12)	0.0029 (11)	0.0050 (11)
C7	0.0363 (16)	0.0233 (16)	0.0285 (15)	0.0152 (14)	0.0107 (12)	0.0092 (12)
C8	0.0189 (12)	0.0195 (14)	0.0128 (11)	0.0115 (11)	0.0045 (9)	0.0010 (10)
C9	0.0164 (12)	0.0229 (15)	0.0167 (12)	0.0088 (11)	0.0047 (9)	0.0031 (10)
C10	0.0194 (13)	0.0294 (17)	0.0219 (13)	0.0067 (12)	0.0080 (10)	0.0024 (12)
C11	0.0331 (15)	0.0237 (16)	0.0238 (14)	0.0114 (13)	0.0145 (12)	0.0062 (12)
C12	0.0312 (15)	0.0305 (17)	0.0207 (13)	0.0184 (13)	0.0087 (11)	0.0088 (12)
C13	0.0205 (12)	0.0231 (15)	0.0208 (13)	0.0118 (12)	0.0034 (10)	0.0026 (11)
C14	0.0195 (12)	0.0186 (14)	0.0153 (12)	0.0116 (11)	−0.0042 (9)	−0.0023 (10)
C15	0.0194 (13)	0.0294 (16)	0.0195 (13)	0.0150 (12)	−0.0045 (10)	−0.0036 (11)
C16	0.0282 (15)	0.0392 (19)	0.0220 (14)	0.0218 (14)	−0.0030 (11)	−0.0063 (13)
C17	0.0373 (17)	0.0298 (18)	0.0300 (16)	0.0230 (15)	−0.0133 (13)	−0.0137 (13)
C18	0.0277 (14)	0.0215 (16)	0.0280 (15)	0.0124 (13)	−0.0113 (12)	−0.0032 (12)
C19	0.0205 (13)	0.0206 (15)	0.0200 (13)	0.0096 (11)	−0.0061 (10)	−0.0003 (11)
C20	0.0133 (11)	0.0181 (14)	0.0154 (11)	0.0075 (10)	0.0004 (9)	0.0046 (10)
C21	0.0184 (12)	0.0218 (15)	0.0228 (13)	0.0089 (11)	0.0009 (10)	0.0031 (11)
C22	0.0122 (12)	0.0233 (16)	0.0398 (16)	0.0023 (12)	−0.0016 (11)	0.0040 (13)
C23	0.0169 (12)	0.0361 (18)	0.0324 (15)	0.0150 (13)	0.0067 (11)	0.0128 (13)
C24	0.0224 (13)	0.0274 (16)	0.0215 (13)	0.0158 (12)	0.0053 (10)	0.0067 (11)
C25	0.0178 (12)	0.0195 (14)	0.0181 (12)	0.0092 (11)	0.0013 (10)	0.0022 (10)
C26	0.0218 (12)	0.0177 (14)	0.0114 (11)	0.0074 (11)	−0.0008 (9)	0.0014 (10)
C27	0.0147 (11)	0.0161 (13)	0.0116 (11)	0.0067 (10)	−0.0034 (9)	−0.0019 (9)
C28	0.0197 (12)	0.0234 (15)	0.0189 (13)	0.0113 (12)	−0.0036 (10)	−0.0018 (11)
C29	0.0196 (13)	0.0307 (17)	0.0227 (13)	0.0169 (12)	−0.0033 (10)	−0.0038 (12)
C30	0.0232 (13)	0.0290 (16)	0.0217 (13)	0.0206 (13)	−0.0051 (10)	−0.0088 (11)
C31	0.0284 (14)	0.0128 (14)	0.0266 (14)	0.0134 (12)	−0.0099 (11)	−0.0029 (11)
C32	0.0396 (17)	0.0153 (15)	0.0301 (15)	0.0108 (13)	−0.0123 (12)	0.0031 (12)
C33	0.0317 (15)	0.0212 (16)	0.0235 (14)	0.0035 (13)	−0.0053 (12)	0.0094 (12)
F1	0.0260 (10)	0.0532 (15)	0.0464 (12)	0.0076 (10)	0.0116 (9)	0.0157 (11)
F1*	0.047 (5)	0.046 (5)	0.068 (5)	0.031 (4)	0.021 (5)	0.017 (5)
F2	0.0554 (15)	0.0489 (15)	0.0332 (11)	0.0388 (13)	0.0103 (10)	0.0054 (10)
F2*	0.043 (5)	0.045 (5)	0.053 (5)	0.022 (5)	−0.002 (5)	−0.001 (5)
F3	0.0747 (17)	0.0305 (13)	0.108 (2)	0.0315 (13)	0.0466 (15)	0.0295 (13)
F3*	0.047 (5)	0.053 (5)	0.058 (5)	0.019 (4)	0.011 (4)	0.005 (4)
F4	0.0367 (13)	0.079 (2)	0.0557 (14)	0.0381 (14)	−0.0208 (11)	−0.0360 (14)
F4*	0.043 (5)	0.049 (5)	0.044 (5)	0.018 (5)	−0.004 (5)	0.001 (5)
B1	0.0262 (15)	0.0273 (17)	0.0447 (18)	0.0170 (13)	0.0062 (13)	0.0046 (14)

### [(1,2,5,6-η)-Cycloocta-1,5-diene](1-methyl-3-propylimidazol-2-ylidene-κC)(triphenylphosphane-κP)iridium(I) tetrafluoridoborate Geometric parameters (Å, º)

**Table:**

Ir1—P1	2.3190 (6)	C16—C17	1.384 (5)
Ir1—C1	2.045 (2)	C17—H17	0.9500
Ir1—C26	2.201 (2)	C17—C18	1.388 (4)
Ir1—C27	2.188 (2)	C18—H18	0.9500
Ir1—C30	2.192 (3)	C18—C19	1.383 (4)
Ir1—C31	2.183 (3)	C19—H19	0.9500
P1—C8	1.826 (3)	C20—C21	1.395 (4)
P1—C14	1.825 (3)	C20—C25	1.392 (4)
P1—C20	1.842 (2)	C21—H21	0.9500
N1—C1	1.364 (3)	C21—C22	1.400 (4)
N1—C2	1.375 (4)	C22—H22	0.9500
N1—C4	1.455 (4)	C22—C23	1.388 (4)
N2—C1	1.357 (3)	C23—H23	0.9500
N2—C3	1.391 (3)	C23—C24	1.381 (4)
N2—C5	1.465 (3)	C24—H24	0.9500
C2—H2	0.9500	C24—C25	1.396 (3)
C2—C3	1.343 (4)	C25—H25	0.9500
C3—H3	0.9500	C26—H26	1.0000
C4—H4A	0.9800	C26—C27	1.402 (3)
C4—H4B	0.9800	C26—C33	1.507 (4)
C4—H4C	0.9800	C27—H27	1.0000
C5—H5A	0.9900	C27—C28	1.517 (3)
C5—H5B	0.9900	C28—H28A	0.9900
C5—C6	1.524 (3)	C28—H28B	0.9900
C6—H6A	0.9900	C28—C29	1.533 (4)
C6—H6B	0.9900	C29—H29A	0.9900
C6—C7	1.527 (4)	C29—H29B	0.9900
C7—H7A	0.9800	C29—C30	1.514 (4)
C7—H7B	0.9800	C30—H30	1.0000
C7—H7C	0.9800	C30—C31	1.398 (4)
C8—C9	1.401 (3)	C31—H31	1.0000
C8—C13	1.399 (3)	C31—C32	1.524 (4)
C9—H9	0.9500	C32—H32A	0.9900
C9—C10	1.388 (4)	C32—H32B	0.9900
C10—H10	0.9500	C32—C33	1.515 (4)
C10—C11	1.381 (4)	C33—H33A	0.9900
C11—H11	0.9500	C33—H33B	0.9900
C11—C12	1.388 (4)	F1—B1	1.369 (4)
C12—H12	0.9500	F1*—B1	1.319 (10)
C12—C13	1.392 (4)	F2—B1	1.409 (4)
C13—H13	0.9500	F2*—B1	1.271 (11)
C14—C15	1.399 (4)	F3—B1	1.396 (4)
C14—C19	1.405 (4)	F3*—B1	1.416 (10)
C15—H15	0.9500	F4—B1	1.365 (4)
C15—C16	1.392 (4)	F4*—B1	1.489 (10)
C16—H16	0.9500		
			
C1—Ir1—P1	91.96 (6)	C16—C17—H17	119.9
C1—Ir1—C26	84.86 (10)	C16—C17—C18	120.2 (3)
C1—Ir1—C27	92.75 (9)	C18—C17—H17	119.9
C1—Ir1—C30	165.69 (10)	C17—C18—H18	119.9
C1—Ir1—C31	155.65 (11)	C19—C18—C17	120.1 (3)
C26—Ir1—P1	167.32 (7)	C19—C18—H18	119.9
C27—Ir1—P1	155.41 (7)	C14—C19—H19	119.7
C27—Ir1—C26	37.25 (9)	C18—C19—C14	120.5 (3)
C27—Ir1—C30	80.04 (9)	C18—C19—H19	119.7
C30—Ir1—P1	89.75 (7)	C21—C20—P1	122.4 (2)
C30—Ir1—C26	96.36 (10)	C25—C20—P1	118.19 (18)
C31—Ir1—P1	98.08 (7)	C25—C20—C21	119.4 (2)
C31—Ir1—C26	80.49 (10)	C20—C21—H21	120.2
C31—Ir1—C27	87.29 (10)	C20—C21—C22	119.5 (3)
C31—Ir1—C30	37.27 (11)	C22—C21—H21	120.2
C8—P1—Ir1	108.32 (8)	C21—C22—H22	119.8
C8—P1—C20	103.33 (11)	C23—C22—C21	120.4 (3)
C14—P1—Ir1	114.74 (8)	C23—C22—H22	119.8
C14—P1—C8	104.88 (12)	C22—C23—H23	119.8
C14—P1—C20	104.99 (11)	C24—C23—C22	120.3 (2)
C20—P1—Ir1	119.15 (8)	C24—C23—H23	119.8
C1—N1—C2	110.5 (2)	C23—C24—H24	120.3
C1—N1—C4	124.5 (2)	C23—C24—C25	119.4 (3)
C2—N1—C4	124.9 (2)	C25—C24—H24	120.3
C1—N2—C3	110.9 (2)	C20—C25—C24	120.9 (2)
C1—N2—C5	126.3 (2)	C20—C25—H25	119.5
C3—N2—C5	122.8 (2)	C24—C25—H25	119.5
N1—C1—Ir1	123.13 (19)	Ir1—C26—H26	114.5
N2—C1—Ir1	132.17 (17)	C27—C26—Ir1	70.89 (13)
N2—C1—N1	104.6 (2)	C27—C26—H26	114.5
N1—C2—H2	126.1	C27—C26—C33	125.4 (3)
C3—C2—N1	107.7 (2)	C33—C26—Ir1	108.29 (17)
C3—C2—H2	126.1	C33—C26—H26	114.5
N2—C3—H3	126.9	Ir1—C27—H27	113.2
C2—C3—N2	106.3 (2)	C26—C27—Ir1	71.86 (13)
C2—C3—H3	126.9	C26—C27—H27	113.2
N1—C4—H4A	109.5	C26—C27—C28	125.0 (2)
N1—C4—H4B	109.5	C28—C27—Ir1	113.69 (16)
N1—C4—H4C	109.5	C28—C27—H27	113.2
H4A—C4—H4B	109.5	C27—C28—H28A	109.1
H4A—C4—H4C	109.5	C27—C28—H28B	109.1
H4B—C4—H4C	109.5	C27—C28—C29	112.6 (2)
N2—C5—H5A	109.2	H28A—C28—H28B	107.8
N2—C5—H5B	109.2	C29—C28—H28A	109.1
N2—C5—C6	112.2 (2)	C29—C28—H28B	109.1
H5A—C5—H5B	107.9	C28—C29—H29A	108.9
C6—C5—H5A	109.2	C28—C29—H29B	108.9
C6—C5—H5B	109.2	H29A—C29—H29B	107.7
C5—C6—H6A	109.4	C30—C29—C28	113.3 (2)
C5—C6—H6B	109.4	C30—C29—H29A	108.9
C5—C6—C7	111.2 (2)	C30—C29—H29B	108.9
H6A—C6—H6B	108.0	Ir1—C30—H30	114.2
C7—C6—H6A	109.4	C29—C30—Ir1	109.96 (18)
C7—C6—H6B	109.4	C29—C30—H30	114.2
C6—C7—H7A	109.5	C31—C30—Ir1	71.01 (15)
C6—C7—H7B	109.5	C31—C30—C29	124.9 (2)
C6—C7—H7C	109.5	C31—C30—H30	114.2
H7A—C7—H7B	109.5	Ir1—C31—H31	113.9
H7A—C7—H7C	109.5	C30—C31—Ir1	71.71 (15)
H7B—C7—H7C	109.5	C30—C31—H31	113.9
C9—C8—P1	118.92 (18)	C30—C31—C32	123.8 (2)
C13—C8—P1	122.21 (19)	C32—C31—Ir1	112.39 (19)
C13—C8—C9	118.8 (2)	C32—C31—H31	113.9
C8—C9—H9	119.5	C31—C32—H32A	109.0
C10—C9—C8	121.0 (2)	C31—C32—H32B	109.0
C10—C9—H9	119.5	H32A—C32—H32B	107.8
C9—C10—H10	120.2	C33—C32—C31	112.7 (2)
C11—C10—C9	119.6 (2)	C33—C32—H32A	109.0
C11—C10—H10	120.2	C33—C32—H32B	109.0
C10—C11—H11	119.9	C26—C33—C32	113.3 (2)
C10—C11—C12	120.2 (3)	C26—C33—H33A	108.9
C12—C11—H11	119.9	C26—C33—H33B	108.9
C11—C12—H12	119.7	C32—C33—H33A	108.9
C11—C12—C13	120.5 (2)	C32—C33—H33B	108.9
C13—C12—H12	119.7	H33A—C33—H33B	107.7
C8—C13—H13	120.1	F1—B1—F2	108.2 (3)
C12—C13—C8	119.8 (2)	F1—B1—F3	107.9 (3)
C12—C13—H13	120.1	F1*—B1—F3*	109.2 (8)
C15—C14—P1	122.3 (2)	F1*—B1—F4*	104.7 (8)
C15—C14—C19	118.6 (3)	F2*—B1—F1*	121.7 (9)
C19—C14—P1	118.6 (2)	F2*—B1—F3*	112.3 (9)
C14—C15—H15	119.8	F2*—B1—F4*	107.0 (9)
C16—C15—C14	120.5 (3)	F3—B1—F2	106.4 (3)
C16—C15—H15	119.8	F3*—B1—F4*	99.1 (7)
C15—C16—H16	120.0	F4—B1—F1	110.9 (3)
C17—C16—C15	120.0 (3)	F4—B1—F2	110.5 (3)
C17—C16—H16	120.0	F4—B1—F3	112.7 (3)
			
Ir1—P1—C8—C9	21.8 (2)	C8—C9—C10—C11	1.1 (4)
Ir1—P1—C8—C13	−155.9 (2)	C9—C8—C13—C12	−1.0 (4)
Ir1—P1—C14—C15	−111.35 (19)	C9—C10—C11—C12	−2.6 (4)
Ir1—P1—C14—C19	60.7 (2)	C10—C11—C12—C13	2.2 (4)
Ir1—P1—C20—C21	−119.42 (19)	C11—C12—C13—C8	−0.4 (4)
Ir1—P1—C20—C25	59.2 (2)	C13—C8—C9—C10	0.7 (4)
Ir1—C26—C27—C28	−106.7 (2)	C14—P1—C8—C9	−101.2 (2)
Ir1—C26—C33—C32	39.3 (3)	C14—P1—C8—C13	81.2 (2)
Ir1—C27—C28—C29	11.2 (3)	C14—P1—C20—C21	10.8 (2)
Ir1—C30—C31—C32	−105.2 (3)	C14—P1—C20—C25	−170.58 (19)
Ir1—C31—C32—C33	13.7 (3)	C14—C15—C16—C17	0.1 (4)
P1—C8—C9—C10	−177.1 (2)	C15—C14—C19—C18	1.1 (3)
P1—C8—C13—C12	176.6 (2)	C15—C16—C17—C18	1.3 (4)
P1—C14—C15—C16	170.79 (19)	C16—C17—C18—C19	−1.5 (4)
P1—C14—C19—C18	−171.26 (19)	C17—C18—C19—C14	0.3 (4)
P1—C20—C21—C22	177.4 (2)	C19—C14—C15—C16	−1.2 (4)
P1—C20—C25—C24	−177.21 (19)	C20—P1—C8—C9	149.1 (2)
N1—C2—C3—N2	−0.4 (3)	C20—P1—C8—C13	−28.6 (2)
N2—C5—C6—C7	168.4 (2)	C20—P1—C14—C15	115.9 (2)
C1—N1—C2—C3	−0.3 (3)	C20—P1—C14—C19	−72.0 (2)
C1—N2—C3—C2	1.0 (3)	C20—C21—C22—C23	0.0 (4)
C1—N2—C5—C6	117.0 (3)	C21—C20—C25—C24	1.5 (4)
C2—N1—C1—Ir1	177.28 (17)	C21—C22—C23—C24	1.1 (4)
C2—N1—C1—N2	0.9 (3)	C22—C23—C24—C25	−0.8 (4)
C3—N2—C1—Ir1	−177.09 (18)	C23—C24—C25—C20	−0.5 (4)
C3—N2—C1—N1	−1.1 (3)	C25—C20—C21—C22	−1.2 (4)
C3—N2—C5—C6	−62.8 (3)	C26—C27—C28—C29	94.9 (3)
C4—N1—C1—Ir1	−5.1 (3)	C27—C26—C33—C32	−39.8 (3)
C4—N1—C1—N2	178.5 (2)	C27—C28—C29—C30	−31.2 (3)
C4—N1—C2—C3	−177.9 (2)	C28—C29—C30—Ir1	35.9 (3)
C5—N2—C1—Ir1	3.1 (4)	C28—C29—C30—C31	−44.4 (4)
C5—N2—C1—N1	179.0 (2)	C29—C30—C31—Ir1	101.5 (3)
C5—N2—C3—C2	−179.2 (2)	C29—C30—C31—C32	−3.6 (4)
C8—P1—C14—C15	7.4 (2)	C30—C31—C32—C33	96.1 (3)
C8—P1—C14—C19	179.41 (18)	C31—C32—C33—C26	−35.7 (3)
C8—P1—C20—C21	120.4 (2)	C33—C26—C27—Ir1	99.3 (2)
C8—P1—C20—C25	−60.9 (2)	C33—C26—C27—C28	−7.4 (4)

### [(1,2,5,6-η)-Cycloocta-1,5-diene](1-methyl-3-propylimidazol-2-ylidene-κC)(triphenylphosphane-κP)iridium(I) tetrafluoridoborate Hydrogen-bond geometry (Å, º)

**Table:**

*D*—H···*A*	*D*—H	H···*A*	*D*···*A*	*D*—H···*A*
C3—H3···F4^i^	0.95	2.38	3.100 (4)	133
C5—H5*A*···F1^ii^	0.99	2.46	3.265 (3)	138
C7—H7*B*···F1*^ii^	0.98	2.53	3.263 (13)	132
C10—H10···F2*^ii^	0.95	2.31	3.073 (16)	136
C19—H19···F3	0.95	2.47	3.413 (4)	171
C19—H19···F3*	0.95	2.53	3.272 (16)	135
C28—H28*B*···F4*^ii^	0.99	2.37	3.292 (18)	154

Symmetry codes: (i) *x*, *y*+1, *z*; (ii) *x*+1, *y*+1, *z*.
